# Moderate Wine Consumption and Health: A Narrative Review

**DOI:** 10.3390/nu15010175

**Published:** 2022-12-30

**Authors:** Silvana Hrelia, Laura Di Renzo, Luigi Bavaresco, Elisabetta Bernardi, Marco Malaguti, Attilio Giacosa

**Affiliations:** 1 Department for Life Quality Studies, University of Bologna, 47921 Rimini, Italy; 2 Section of Clinical Nutrition and Nutrigenomics, Department of Biomedicine and Prevention, University of Tor Vergata, 00133 Rome, Italy; 3 Department of Sustainable Crop Production—Viticulture and Pomology Section, Università Cattolica del Sacro Cuore, 29122 Piacenza, Italy; 4 Department of Biosciences, Biotechnologies and Biopharmaceutics, University of Bari “Aldo Moro”, 70121 Bari, Italy; 5 Department of Gastroenterology and Clinical Nutrition, Policlinico di Monza, 20900 Monza, Italy

**Keywords:** wine consumption, alcohol consumption, Mediterranean diet, resveratrol, grape polyphenols

## Abstract

Although it is clearly established that the abuse of alcohol is seriously harmful to health, much epidemiological and clinical evidence seem to underline the protective role of moderate quantities of alcohol and in particular of wine on health. This narrative review aims to re-evaluate the relationship between the type and dose of alcoholic drink and reduced or increased risk of various diseases, in the light of the most current scientific evidence. In particular, in vitro studies on the modulation of biochemical pathways and gene expression of wine bioactive components were evaluated. Twenty-four studies were selected after PubMed, Scopus and Google Scholar searches for the evaluation of moderate alcohol/wine consumption and health effects: eight studies concerned cardiovascular diseases, three concerned type 2 diabetes, four concerned neurodegenerative diseases, five concerned cancer and four were related to longevity. A brief discussion on viticultural and enological practices potentially affecting the content of bioactive components in wine is included. The analysis clearly indicates that wine differs from other alcoholic beverages and its moderate consumption not only does not increase the risk of chronic degenerative diseases but is also associated with health benefits particularly when included in a Mediterranean diet model. Obviously, every effort must be made to promote behavioral education to prevent abuse, especially among young people.

## 1. Introduction

All scientific medical literature clearly underlines that the abuse of alcohol is seriously harmful to health. These findings have led many health organizations and institutions responsible for disease prevention and health defense to suggest measures aimed at favoring the strict containment of alcohol consumption until the eventual achievement of the zero alcohol target [1]. However, much epidemiological and clinical evidence seem to underline the protective role of moderate quantities of alcohol and in particular of wine on health [2].

This narrative review aims to re-evaluate the relationship between the type of alcoholic drink and the dose and the reduction or increase in the risk of various diseases, in the light of the most current scientific evidence. In particular, the correlation with cardiovascular pathologies, type 2 diabetes, neurological degenerative diseases and longevity was analyzed. Furthermore, particular attention was paid to the highly debated chapter on the correlation between alcohol/wine and cancer risk. The differences between wine and other alcoholic beverages were analyzed and the modulation of biochemical pathways and gene expression of wine bioactive components were carefully evaluated. This study provides scientific data to tailor the efforts in educating clinicians and the public about the relationship between wine consumption and favorable disease outcomes.

The main objective of this narrative review is to provide concrete scientific evidence to guide the institutions that must establish guidelines on the correct and conscious consumption of alcoholic beverages and in particular of wine, in order not to damage health and indeed possibly to protect it and reduce the risk of various diseases.

## 2. Literature Search Strategy

The literature search was initially conducted on scientific literature databases (PubMed, Scopus, Google Scholar and Web of Science) focusing on the following MeSH terms: moderate alcohol consumption, wine polyphenols, trans-resveratrol, antioxidant activity, gene expression, biochemical pathways, Mediterranean diet, drinking pattern. Peer-reviewed papers had to be written in English, available in full text, and published mainly between 2010 and 2022. In addition, papers published before 2010 were included if they were relevant in the field. We searched for research articles, reviews or editorials. Many articles were obtained, some of which did not precisely meet the field of interest; therefore, screening was undertaken by four independent authors. Disagreement was resolved by discussion and, if required, by a fifth independent author.

**Figure 1:**
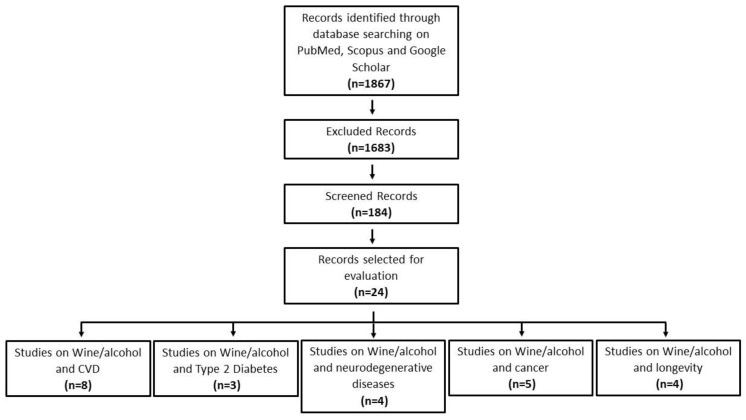
Flowchart of literature search on epidemiological studies.

Epidemiological studies selection was performed according to Egger et al. [3] following these steps:

1. Configuration of the working group: two operators, experts in clinical epidemiology (one acting as a methodological operator and one participating as a clinical operator).

2. Formulation of the questions based on the considerations indicated in the abstract: the correlation of moderate alcohol and wine consumption and risk of various diseases (cardiovascular diseases, type 2 diabetes, neurodegenerative diseases, cancer) and longevity).

3. Recognition of the relevant studies: research was carried out on PubMed, Scopus and Google Scholar as follows: (a) definition of the keywords (alcohol, wine, cardiovascular disease, type 2 diabetes, neurodegenerative diseases, cancer, longevity), inserting the interest field of the documents to be searched, grouped in quotation marks (“…”), and used separately or in combination; (b) use of the Boolean variable (true or false) AND operator, that allows for the establishment of logical relations among concepts; (c) research modalities: advanced search; (d) limits: papers published in the last 22 years (also, papers published before 2000 were included if they were relevant in the field); humans; adults; languages: English; (e) manual search performed by the researchers experienced in clinical nutrition through the revision of articles, focusing on alcohol and wine consumption and health risks.

4. Published in journals qualified in the Index Medicus.

5. Analysis and presentation of the outcomes: paragraphs about the correlation between low-to-moderate wine consumption and health, and the data extrapolated from the “revised studies” were collocated in tables; in particular, the author, year of publication and characteristics of the study were specified for each study.

6. An analysis of the reports in the form of a narrative review was carried out and discussed. At the beginning of each section, the type of studies chosen and the keywords considered are reported. Figure 1 indicates the flow chart of the literature search on epidemiological studies.

## 3. Ongoing Research to Improve the Phenolic Profile of Grapes and Wine

Wine contains many bioactive compounds and among them the phenolics (anthocyanins, flavones, flavonols, flavanols, alkylphenols, tyrosols, phenolic acids, lignans, stilbenes) are the most representative. An overview on grapevine phenolic synthesis, role and factors affecting their occurrence in grapes and wine is well reported [4,5]. Among phenolics, stilbenes will be emphasized in this section.

Stilbenes, in soft (fleshy) tissues like berry skins, act as phytoalexins (inducible antifungal compounds) since they are synthesized by the plant in response to pathogen attack; in addition, some abiotic elicitors (like for instance UV irradiations, ozone, fosetyl-Al, methyl jasmonate, chitosan oligomers, etc.) can trigger stilbene synthesis [6,7,8,9,10]. A lot of stilbenic compounds have been detected in grapes and wine (resveratrol, piceid, pterostilbene, viniferins, astringin, piceatannol, resveratrol trimers and tetramers, etc.). Resveratrol is the most studied and best-known stilbene and the production of high resveratrol-containing grapes and wines relies on quality-oriented viticulture (suitable terroirs, sustainable cultural practices) and on wine-making technologies which avoid degradation of the compound.

Grapes’ levels of resveratrol (and its glucoside—piceid) are affected by the grape variety, clone, meteorological conditions, soil type and cultural practices [11,12]. As a rule, the higher the concentration in grapes, the higher the concentration in wine.

Resveratrol is present in ripe grapes of both red and white varieties, being higher in red berries compared to the white ones [13]. Also the clone can play a role, as reported in a pot trial or in the vineyard [14]. Cooler compared to warmer conditions during ripening time increase resveratrol berry concentrations, as does higher vineyard elevation [15].

Increasing nitrogen supply has a negative effect on berry resveratrol levels, while calcium supply (by foliar spray) has a positive effect [16].

Leaf removal in the cluster zone at veraison improves resveratrol values in cooler (during ripening time) years, while in warmer years the opposite occurs.

Cluster thinning improves wine resveratrol concentration and antioxidant capacity, while irrigation (versus non-irrigation) reduces resveratrol concentrations in grapes [17].

It is difficult to compare resveratrol grape concentration data (from the literature) as affected by biotic/abiotic elicitors and viticultural factors, because of different extraction methods and units of measurements.

According to Bavaresco et al. [11] resveratrol is present in a considerable higher amount in red wines than white wines because it is mainly present in the berry skin, and white wines are usually produced with no or limited maceration with the pomaces.

To some extent, oenological practices can also potentially affect resveratrol in wine, such as the yeast species [18,19], the occurrence of malolactic fermentation [20], the time of maceration [21], the levels of fining agents usually added to stabilize the red wines [22] and aging [23,24,25].

In addition, the use of specific postharvest techniques can modulate resveratrol and other stilbenes in grapes; for example, postharvest UV-C irradiated grapes are a potential source for producing stilbene-enriched red wines [26].

The highest wine concentration of total resveratrol, according to the literature, is 36 mg/L.

## 4. Wine Bioactive Compounds: Modulation of Biochemical Pathways and Gene Expression

Wine is a complex hydro-alcoholic solution containing a huge variety of different compounds; most of its phytochemicals have been isolated, identified and studied with the aim of identifying those responsible for a beneficial effect on human health. Wine complexity is extremely difficult to reproduce in vitro; this complexity and the multitude of different components allow the wine to exert synergistic biological effects that are much greater and more heterogeneous than those performed by individual components.

As described by recent systematic reviews, wine phytochemical composition strongly depends on the interaction between environmental factors, such as the “terroir” a term derived from Latin, which defines a specific geographical area, with oenological and grape cultivation applied practices which, altogether, define the unique features of a specific wine. In fact, previous studies have reported that wine bioactive compounds include polyphenols such as flavonoids, (+)-catechin, quercetin and anthocyanins, and stilbenes such as resveratrol (3, 5, 4′-trihydroxystilbene) [27,28,29,30].

The high number of factors influencing phytochemical composition and content leads to a very wide range of total phenolic content. For red wines, it ranges between 1531 and 3192 mg of gallic acid equivalents (GAE) per liter, while it was between 210 and 402 for white wines [31].

Besides phenolic compounds, melatonin has also been identified in wines, and its presence might be responsible for its role in putative wine health benefits [32,33,34,35].

### 4.1. Modulation of Biochemical Pathways

In red wine, quercetin represents the main flavanol, and its concentration is approximatively 50 mg/L. Dietary flavonols’ beneficial effects have been related to their antioxidant properties, to the activation of endogenous antioxidant mechanisms and to the upregulation of nitric oxide synthase (NOS) expression. Moreover, quercetin has been demonstrated to counteract and decrease inflammation; it inhibits the nuclear translocation of nuclear factor kappa-B (NF-kB) and reduces the expression of Toll-like receptors (TLR2 and TLR4) [36].

In vivo, most polyphenols are poorly bioavailable and largely biotransformed; for these reasons doubts have been raised about the fact that the above-mentioned antioxidant and anti-inflammatory activities can exert a primary role in in vivo polyphenol effects; however, they can modulate gene expression and intracellular signaling cascades involved in cell survival and protection. In fact, in vivo, polyphenols show a weak, even though effective, direct antioxidant effect [37]. From a kinetic perspective, they do not act as free radical scavengers, and considering their poor bioavailability their contribution to the cellular antioxidant system is very little. Moreover, metabolites as well as parental compounds can exert biological effects and the identification of microbiota-synthesized metabolites greatly broadens the list of compounds that can exert a biological effect [38].

Even though in vivo wine polyphenols should not be considered as direct antioxidants, they might behave as such in a few conditions. During digestion, in the stomach, especially when red meat is consumed, lipid peroxides are produced up to mM concentration [39,40]. Such peroxide formation can be prevented and reduced by the presence of foods and beverages such as extra virgin olive oil and red wine which are rich in polyphenols that behave as direct antioxidants and scavenge lipid peroxides.

Nowadays, wine bioactive components are often defined as “interactive biomolecular vehicles”, or rather molecules able to protect the organism against a variety of stress-related damages by modulating intercellular signaling pathways leading to the inhibition of pro-inflammatory molecule synthesis, ROS formation and nuclear damage, and by inducing antioxidant enzyme gene expression. Particularly in the case of resveratrol, but also of other wine polyphenols and bioactive components, this induction of antioxidant genes involves the activation of nuclear-factor-erythroid-2-related factor 2 (Nrf2) and its downstream pathways via xeno-hormesis. Nrf2 pathway activation modulates multiple aspects of cellular defense against oxidative stress, such as detoxification, cell metabolism and cell proliferation regulation, which are crucial in the pathological mechanism of various diseases [41].

It is now widely recognized and demonstrated that Nrf2 is crucial in the ARE-mediated gene expressions of redox enzymes, including antioxidants and detoxicant. Therefore, the Nrf2 pathway is essential to prevent those diseases whose pathophysiological basis is represented by oxidative stress and inflammation [42,43]. Thus, the Nrf2 pathway may be a potential option for preventing chronic/degenerative diseases.

Polyphenolic compounds typically found in red wine, such as quercetin, catechin, epicatechin, procyanidin B2, piceatannol and silibinin, have been demonstrated to counteract against oxidative stress by activating the Nrf2 pathway and by upregulating a series of antioxidant enzymes and proteins GSH, GST, GSTP1, NQO-1, HO-1 and SOD, and metallothioneins 1 and 2 (MT-1/2) [44]. In addition, the Nrf2/ARE pathway regulates the expression of over 500 genes including phase I and II detoxification enzymes, proteasome subunits, chaperones, growth factors, and other transcription factors and transport proteins [45].

Among all the phyto-components found in wine, resveratrol, belonging to the stilbene family, is certainly the most studied one (for a comprehensive review see [46]).

**Figure 2:**
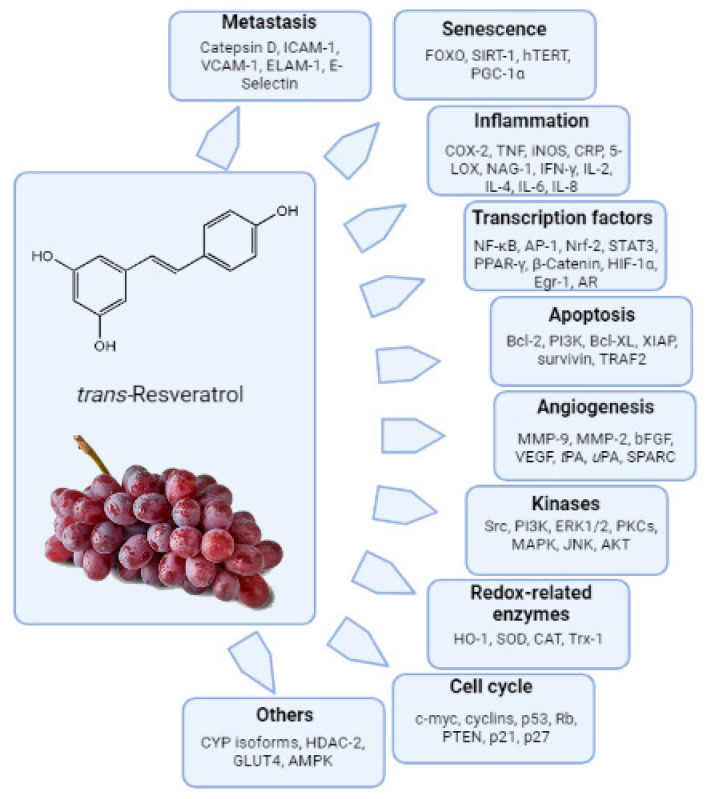
Resveratrol-modulated biochemical targets. AP-1, activator protein 1; CAT, catalase; CDK, cyclin dependent kinase; COX, cyclooxygenase; CRP, C-reactive protein; CYP, cytochrome P450; ER, estrogen receptor; ERK, extra cellularly regulated kinase; GPx, glutathione peroxidase; HIF, hypoxia inducing factor; hTERT, human telomerase reverse transcriptase; ICAM, intracellular adhesion molecule; IAP, inhibitor of apoptosis proteins; iNOS, inducible nitric oxide; MAPK, mitogen activated protein kinase; MMP, matrix metalloproteinase; NFκB, nuclear factor kappa B; PI3K, phosphoinositide-3 kinase; Rb, retinoblastoma; SOD, superoxide dismutase; STAT, signal transducer and activator of transcription; SPARC, secreted protein acidic and rich in cysteine; TNF, tumor necrosis factor; VEGF, vascular endothelial growth factor.

Many in vitro studies have demonstrated significant biological effects and evidenced that trans-resveratrol (trans-3,5,4′-trihydoxystilbene, RES, Figure 2) modulates various targets related to different biochemical pathways. The interest in resveratrol has been, and still is, aimed both at its potential application as a therapeutic agent and as a protective agent against numerous diseases. Studies using purified enzymes and cell cultures have suggested that resveratrol has antioxidant, anti-inflammatory, anti-aging and anti-carcinogenic properties that might be relevant to chronic disease prevention/counteraction and/or longevity in humans [47,48].

Describing all biologically relevant resveratrol targets is a very complex task, made more difficult by conflicting results obtained in different systems and by the uncertainty of whether effects are either direct or indirect. A full discussion of these issues is beyond the scope of this review and has been attempted by other authors, e.g., [49,50].

More recently, a trans-resveratrol dimer named trans-epsilon-viniferin (ε-viniferin), has attracted the interest of scientists in the field of human health [51]. Its main dietary source is represented by red wine, and the highest concentrations observed were about 1 mg/L [52].

In the last fifteen years, many studies have investigated ε-viniferin biological effects, and its antioxidant and anti-inflammatory properties have been described (for a review see [51]). In some cases, ε-viniferin biological properties have been reported to be even greater than those of resveratrol itself. Its beneficial effects in both in vivo and in vitro models of cancers, obesity, obesity-related disorders, and cardiovascular or neurodegenerative diseases have been demonstrated. Moreover, ε-viniferin has also shown effective antimicrobial properties [53].

As with most phytochemicals, including resveratrol, in order to be able to clearly define its potential impact on health, it is essential to define its absorption and metabolism rate [54]. Resveratrol absorption rate is approximately 75% (25 mg oral dose) [55], but the bioavailability is poor [54]. Resveratrol is rapidly metabolized by phase II metabolic enzymes, and its conjugates are quickly absorbed through the gastrointestinal tract and they become detectable in serum 30–60 min after ingestion [56].

Resveratrol cannot be considered the only thing responsible for the benefits associated with moderate red wine consumption, rather these effects are attributable to the entire pool of antioxidants present.

Among the polyphenols, resveratrol has emerged as a key component in the regulation of vascular homeostasis. It has been shown that it interacts with IR-1 and SIRT1, increasing antioxidant capacity, improving metabolic health, and regulating endothelial function through activation and upregulation of eNOS while inhibiting inflammatory pathways [57]. Moreover, as resveratrol activates SIRT1, it could interfere with myocardial fibrosis through the factor β (TGF-β)/Smad2/3 pathway [58], eliminating collagen synthesis and cardiac fibroblast differentiation [59]. Resveratrol can protect from cardiac insufficiency and cardiac fibrosis, induced by high blood pressure, by inhibiting the PTEN/AKT/Smad2/3 and NF-kB signaling pathway, resulting in heart protection. For example, it inhibits cytochrome P450 (CYP) [60].

Furthermore, resveratrol can influence the occurrence and progression of myocardial fibrosis by activating sirtuin 3 (SIRT3), regulating the levels of type I collagen gels (COL1), type III collagen gels (COL3) and hydroxyproline (Hyp), and the expression of TGF-β, or by regulating the levels of SDF-1 and immune cytokines such as TNF-α, malondialdehyde (MDA) and IL-1 in oxidative stress [61]. Particularly, resveratrol results in improving adriamycin-induced myocardial fibrosis by increasing SIRT1 and by decreasing factor β1 (TGFβ1), which lead to an increase of glutathione (GSH) and SOD and a decrease of MDA, Hyp, TNF-α and creatine kinase-MB (CK-MB).

Resveratrol acts to improve myocardial fibrosis induced by viral myocarditis by decreasing carboxyterminal propeptide of type I procollagen (PICP) and amino-terminal propeptide of type III procollagen (PIIINP), and by increasing aminoterminal propeptide of type I pro-procollagen (PINP).

Moreover, resveratrol interferes with other cytokines, as tissue inhibitor of metalloproteinases-2 (TIMP-2) and matrix metalloproteinase-2 (MMP-2), an important enzyme related to the MMP system that regulates myocardial matrix metabolism, plays a pivotal role in matrix degradation and collagen synthesis and mediates the activation of several cytokines, such as TNF-α, TGF-β and IL-1 [62]. In particular, resveratrol increases TIMP-2 and decreases MMP-2 and thereby regulates the MMP-2/TIMP-2 ratio, leading to an improvement of atherosclerosis-induced myocardial fibrosis. The coordinated reduction of MMP-2, MMP-9 and fibroblast growth factor 21 (FGF21) incurred by resveratrol results in an advance of myocardial fibrosis induced by alcohol.

Resveratrol also regulates miRNAs in I/R. It is able to regulate the extracellular-signal-regulated kinase (ERK), mitogen-activated protein (MAP) kinase signaling pathway in cardiac fibroblasts through miR-21, miR-20b, miR-27a and miR-9 [63]. The coordinated decrement incurred by resveratrol of diacylglycerol (DAG)/protein kinase A (PKA) and ROS/ERK/TGFβ1 pathways that leads to a reduction of periostin results in an improvement of myocardial fibrosis induced by diabetes.

### 4.2. Wine Intake and Gene Expression

Wine, being a hydro-alcoholic solution, provides some alcohol to the diet, and alcohol, even at a low intake, may exert harmful effects on health.

Among others, its chronic consumption is directly related to numerous non-communicable diseases, such as liver cirrhosis and cancers. Alcohol is classified, by IARC, within Group 1 of carcinogens; it is causally associated with the development of cancers of the upper digestive tract and liver, and can be positively associated with colorectum and female breast cancer with sufficient evidence. During its metabolism, alcohol is oxidized to acetaldehyde. Acetaldehyde is a reactive substance and it might mediate most ethanol-induced toxicity leading to chronic disease development, such as liver and cardiovascular diseases, and cancer [64]. Chemically, acetaldehyde shows electrophilic behavior; it can directly interact with proteins, lipids and DNA to form covalent conjugates [65].

These conjugates might affect cellular homeostasis and survival through protein structure alteration and/or DNA damage. Aldehyde dehydrogenases (ALDHs) represent an enzyme superfamily composed of 19 members that play a key role in the metabolism of aldehydes by catalyzing aldehyde oxidation. Among the ALDH family, ALDH class-2 (ALDH2) is a mitochondrial isoenzyme and it is mainly expressed in the liver. It plays a major role in acetaldehyde oxidation into nontoxic acetic acid [66].

Two quercetin metabolites, namely 3-hydroxyphenylacetic acid (OPAC) and 3,4-dihydroxyphenylacetic acid (DOPAC), have been demonstrated to induce drug metabolic enzymes. Moreover, OPAC showed ALDH-enhancing activity in a cultured hepatocyte model [67]; in this study OPAC enhanced total ALDH activity by upregulating gene transcription through the aryl hydrocarbon receptor (AhR)-dependent pathway. Due to its unique biological properties, OPAC may potentiate acetaldehyde metabolism by ALDHs, and also contribute to protection from alcohol-induced liver injury after the consumption of quercetin-rich diets.

If OPAC appears as the most effective metabolite in promoting acetaldehyde detoxification via ALDHs, other nutraceutical bioactive compounds might contribute to a protective strategy aimed to keep low acetaldehyde levels. Resveratrol and flavonoids from red wine have been demonstrated to modulate the expression of gamma-glutamylcysteine synthetase, the rate limiting enzyme in the synthesis of glutathione (GSH), the most important endogenous cellular antioxidant which is involved both in cellular antioxidant defenses and xenobiotic detoxification [68]. GSH directly participates in acetaldehyde detoxification, and GSH levels decrease in the presence of acetaldehyde [69].

Oxidative stress, inflammation, lipid and carbohydrate profiles, and drug metabolism can affect gene expression. Several studies show that beverages and foods characterized by a significant antioxidant effect contain substances capable of preventing damage to the cellular system due to ROS. One of the biochemical mechanisms responsible for the aging process is the progressive loss of perfect DNA replication in the daughter cells. After several replication processes, these alterations accumulate in the transcription of the cellular DNA and activate gene sequences which can cause degeneration and death in the long term. Some nutraceuticals could reduce these replication errors by modulating the related inflammatory processes and counteracting ROS effect. ROS, which increase in the postprandial state, have been reported to be able to damage cellular structures and activate some transcription factors involved in the activation of the gene expression related to immunity, inflammation, cell proliferation, growth and apoptosis [70,71]. In this way, ROS accelerate aging and lead to cardiovascular, neurodegenerative and oncological diseases [72]. Furthermore, they are responsible for the oxidation of LDL-C (Ox-LDL), which accumulate in the tunica intima, where they are swallowed by macrophages [73].

Inflammation induced by oxidative stress and Ox-LDL on vascular cells, by increasing the adhesion of monocytes and macrophages, leads to the formation and infiltration of cholesterol-laden foam cells into the vessel wall, leading to the development of the atherosclerotic process [73,74].

Polyphenols present in red wine, such as phenolic acids, stilbenes, tannins and flavonoids, such as catechin, quercetin and anthocyanins, can potentiate the endogenous antioxidant system of consumers [75].

Following the intake of red wine, the endogenous antioxidant defense systems are activated [76], such as the activities of superoxide dismutase (SOD), catalase (CAT) and glutathione peroxidase (GPx) [77,78]. The reduction of circulating ROS levels, and consequently of the LDL oxidation process [79], depends on the expression levels of SOD and GPx. Indeed, SOD can convert the superoxide anion (O-) into hydrogen peroxide (H_2_O_2_), reducing O-levels and mitochondrial DNA damage [80]; H_2_O_2_ will be transformed into water by GPX1 or CAT [78]. CAT, SOD and GPX increase their expression by the dietary supplementation of resveratrol and quercetin [81].

The Mediterranean diet, which is rich in vegetables, legumes, fruits and nuts, cereals, fish, olive oil, and a moderate amount of wine during meals, with lower amounts of meat and dairy products, is recommended as a healthy dietary pattern. Besides wine, in the MedDiet bioactive components from other foods, such as polyphenols and phytosterols from olive oil (hydroxytyrosol, tyrosol, oleocanthal), nuts, fruits and vegetables (flavonoids, mainly), can contribute to increasing the protective effects through synergic mechanisms.

Some benefits of the Mediterranean-diet-related wine consumption may be related to the pattern of consumption rather than simply the quantity of alcohol consumed. The Mediterranean alcohol-drinking pattern, the so called “Mediterranean way of drinking” that means a moderate wine intake mainly with meals [2], could represent the best way to decrease the toxic effects of ethanol and simultaneously increase the antioxidant/detoxifying defenses thanks to the synergic effect of a wide range of bioactive components able to modulate the body’s defenses and protect against chronic/degenerative diseases.

Di Renzo et al. [79] highlighted the effect of combined intake of red (RW) or white wine (WW) or vodka (VDK) with a Mediterranean meal (MeDM) or high-fat meal (HFM) in a clinical study conducted on 54 healthy volunteers. They examined the Ox-LDL and evaluated the gene expression of selected genes belonging to the inflammatory and oxidative stress pathway, such as CAT, SOD2 and GPX1. Therefore, a controlled randomized clinical trial was performed on healthy volunteers in fasting status or postprandial time, after a Mediterranean or a high-fat meal, with or without alcoholic beverage intake. Notably, they observed a significant upregulation of CAT only after RW. Conversely, WW and VDK administration determined a significant downregulation of CAT gene expression as well as the combination of HFM with WW and VDK, showing a possible downstream decrement of antioxidant enzyme gene expression caused by beverages with low or null polyphenolic concentrations. The expression of the SOD2 gene was upregulated in WW, MeDM + VDK treatment, and especially in RW administration, demonstrating a greater sensitivity of this gene to high and low polyphenolic content. On the other hand, HFM + VDK treatment determined a downregulation of its expression.

Furthermore, it has been demonstrated that red wine polyphenols can also regulate inflammation, reducing the risk of related diseases [36]. Inflammation is a complex biological process characterized by the coordinated regulation of different sets of genes, such as chemokine C-C motif ligand 5 (CCL5). CCL5 is a chemotactic cytokine, generally called RANTES (regulated on activation, normal T cell expresser and secreted), which plays several roles in inflammatory disease [82]. Moreover, in humans, seven types of sirtuins, regulators of silent information (SIR), have been identified [83]. Sirtuins are a class of proteins with deacetylase or monoribosyltransferase activity, which regulate NAD +-dependent deacetylase on different biological processes such as life span, aging, neurodegeneration, tumorigenesis and metabolic diseases. Although its function and biological mechanism are not entirely understood, the cytoplasmic sirtuin protein SIRT2 has been shown to increase in response to oxidative stress. Still, it promotes cell death through Forkhead Box (FOXO) proteins [84].

Di Renzo et al. [85] highlighted the effects of the combined intake of red wine with different meals in a clinical study. Notably, they found that the Ox-LDL levels significantly decreased after a Mediterranean Meal (MM) compared to a McDonald’s Meal (McD) or after a Mediterranean Meal with 250 mL of red wine (MMRW) compared to fasting with red wine (FRW), or after a McDonald’s Meal with red wine compared (McDRW) to McD, indicating that the antioxidant potential of the bioactive compounds found in red wine and the Mediterranean diet may be an essential component of a holistic approach to combatting LDL oxidation.

Moreover, they analyzed the variation of gene expression of five genes related to oxidative stress and inflammation depending on the consumption of different meals with and without red wine.

They observed that CAT expression decreased significantly after McD. On the contrary, a significant increase in CAT expression was observed in FRW compared to the baseline (B) and in McDRW compared to the ones without wine. At the same time, GPX1 expression increased significantly in FRW, McDRW and MMRW compared to B. No significant SOD expression was observed in all conditions. CCL5 expression significantly increased in the comparison between McD, MM and both meals with wine versus B, and in FRW versus McDRW. Meanwhile, CCL5 expression significantly decreased between MM versus the ones with red wine.

SIRT2 expression also increased significantly in comparison to FRW versus MMRW. Moreover, after McD with and without wine consumption, SIRT2 is expressed to a lesser extent than after MM, demonstrating that SIRT2 is upregulated in response to oxidative stress and caloric restriction and promotes cell death under severe stress conditions via interaction with FOXO3a [86]. Furthermore, after an MMRW, it seems that a higher expression of SIRT2 was negatively correlated with the expression of CCL5, confirming a protective effect of transduced PEP-1-SIRT2 against inflammation and oxidative stress. A positive correlation between SIRT2 and CAT was also observed, which may be due to SIRT2 increasing expression of CAT, confirming that modulation of SIRT2 through the diet may have a significant impact on inflammation [87].

Several studies have shown that many of the polyphenols present in red wine can have different physiological effects by activating key signaling pathways such as the insulin receptor 1 (IR-1) and sirtuin 1 (SIRT1), which are involved in insulin sensitivity, cellular regulation and inflammation processes [88].

## 5. Wine and Health: Epidemiological Data

The correlation between wine consumption and health has been the subject of a long-standing debate. The low-to-moderate consumption of wine has been shown to be associated with various health advantages in both male and female populations. As previously discussed, the beneficial effects of wine are mostly derived from its polyphenolic content [59] and this represents the crucial difference between wine and other alcoholic beverages. As a matter of fact, red wine contains on average 1.8 g/L of polyphenols, and their content in white wine ranges between 0.2 and 0.3 g/L [89,90,91], while their content in beer averages 28 mg/100 mL and spirits hardly do not contain any. The major aim of this narrative review was to consider the correlation between low-to-moderate wine consumption and health.

### 5.1. Low-to-Moderate Wine Consumption and Cardiovascular Diseases

This research was conducted with the keywords: “alcohol” AND “wine” AND “cardiovascular disease (CVD)” AND “ischemic heart disease (IHD)”. We analyzed a total of eight studies: six population-based prospective studies, one multicenter open-label prospective study and one meta-analysis. The description of the studies is presented in Table 1.

In all eight selected studies a reduction in the risk of cardiovascular disease, and in particular of IHD, was observed in subjects with moderate alcohol consumption com-pared to abstainers [92,93,94,95,96,97] (Table 1). In particular, three studies evidenced the beneficial role of wine in the prevention of cardiovascular diseases (CVD) [95,98,99] and one demonstrated that among moderate alcohol drinkers there is an advantage for wine drinkers over non-wine drinkers [95]. As a matter of fact, since 1992, observing the French population, characterized by a diet rich in saturated fat and wine and with high serum cholesterol levels, but with a CVD mortality significantly lower than other Western populations, the role of wine compared to other alcoholic beverages in preventing CVD was highlighted [100]. Since then, it has been shown that consuming three to five glasses of red wine per day is more beneficial in reducing CVD risk and overall mortality than other alcoholic beverages, and that wine consumption has an inverse association with CVD, cerebrovascular disease and overall mortality [95,101]. It was also shown that low (1–7 drinks/week) and moderate (8–21 drinks/week) wine drinkers have 20% and 24% less all-cause mortality than non-drinkers of wine, respectively [98]. Furthermore, compared with abstainers, alcohol drinkers with an intake of 5 to 15 g per day were associated with a 26% lower risk of cardiovascular disease, 35% lower risk of total mortality and 51% lower risk of cardiovascular disease mortality, if the alcohol consumption was mostly red wine [102]. Various biochemical mechanisms are involved in the cardioprotective effect of moderate red wine consumption [103]; however, the debate on the real cardioprotective effects of moderate wine consumption is still heated today [104]. Obviously, as far as CVD prevention is concerned, abstainers should not start drinking alcohol to reduce their CVD risk, but the epidemiological evidence indicates that there is no reason to suggest to those who drink wine in moderation to stop drinking it.

**Table 1:** Studies on wine consumption and risk of cardiovascular diseases.

Wine/Alcohol Consumption and CVD Risk	Number of Subjects	Study Design	References
Alcohol consumption is inversely related to coronary heart disease incidence (*p* for trend < 0.001).	51,529 male healthy professionals	Prospective study	Rimm et al., 1991 [92]
Strong negative association between moderate alcohol consumption and the risk of nonfatal myocardial infarction and death from coronary heart disease.	7705 Japanese men living in Hawaii	Cohort study	Yano et al., 1977 [93]
Risk of coronary heart disease decreased from 0 to 20 g/day of alcohol (RR = 0.80; 95% CI: 0.78, 0.83); evidence of a protective effect up to 72 g/day (RR = 0.96; 95% CI: 0.92, 1.00) and increased risk above 89 g/day (RR = 1.05; 95% CI: 1.00, 1.11). Lower protective effects in women and in men living in countries outside the Mediterranean area.	28 cohort studies	Meta-analysis	Corrao et al., 2000 [94]
Compared with non-drinkers, light drinkers who avoid wine have a relative risk for death from coronary heart disease of 0.76 (CI, 0.63 to 0.92) and those who drank wine have a risk of 0.58 (CI, 0.47 to 0.72).	13,064 men and 11,459 women 20 to 98 years of age	Pooled cohort studies	Grønbaek et al., 1995 [95]
IHD-associated mortality: 62 men. In men, RR for IHD in drinkers vs nondrinkers was 0.51 (95% CI, 0.27–0.95). Report a cardioprotective effect from IHD in a predominantly beer drinking population (starts with 0.1–0.99 g/d alcohol intake), and effect did not decrease with higher consumption.	2084 subjects (1071 men; 1013 women)	Population-based prospective study	Keil et al., 1997 [97]
Wine drinkers had lower mortality from IHD than non-wine drinkers (*p* = 0.007). At all levels of intake of alcohol, wine drinkers were at a significantly lower risk for all-cause mortality than nonwine drinkers (*p* < 0.001).	24,525 (13,064 men; 11,459 women)	Population-based prospective cohort	Grønbaek et al., 2000 [98]
For middle-aged women, moderate alcohol consumption decreased the risk of IHD. (Women who consumed 5–14 g alcohol/d had a RR of 0.6; 15–24 g/d RR 0.6; ≥25 g/d RR 0.4.)	87,526 women	Population-based prospective cohort	Stampfer et al., 1988 [96]
Moderate intake of wine was associated with a significant reduction in cardiovascular events including cardiovascular death, non-fatal MI and nonfatal strokes: HR, 0.87 (95% CI, 0.76–0.99). Risk of cardiovascular events was significantly reduced by 13% with wine consumption up to 0.5 L/d (defined as moderate consumption).	11,248 patients with recent myocardial infarction (MI) (9601 men; 1647 women)	Multicenter open-label prospective study	Levantesi et al., 2013 [99]

### 5.2. Low-to-Moderate Wine Consumption and Type 2 Diabetes

This research was conducted with the keywords: “alcohol” AND “wine” AND “type 2 diabetes”. We analyzed a total of three studies: one population-based prospective study, one case–control study and one meta-analysis. The description of the studies is presented in Table 2.

Several studies investigated red wine consumption and its effects on glucose levels and type 2 diabetes (T2D). Two of the selected studies demonstrated that regular red wine drinkers have lower glucose levels [105] and a lower occurrence of diabetes compared to abstainers [106] (Table 2). Furthermore, it is known that red wine consumption reduces plasma insulin and the homeostasis model assessment of insulin resistance [107]. Women with regular red wine consumption were found to have the lowest risk of several diseases such as type T2D [108]. The third selected study, conducted on patients with T2D, showed that those who reported moderate alcohol consumption, mainly of wine, had fewer cardiovascular events and lower all-cause mortality [109] (Table 2). Moreover, 150 mL/day of red wine intake for two years significantly increased HDL-C and Apo AI levels and decreased the total cholesterol (TC)/HDL ratio in patients with T2D [110]. In conclusion, red wine is protective against T2D and is associated with improved insulin sensitivity.

**Table 2:** Studies on wine consumption and type 2 diabetes.

Wine/Alcohol Consumption and Type 2 Diabetes (T2D)	Number of Subjects	Study Design	References
Compared with lifetime abstainers, the relative risk (RR) for type 2 diabetes among men was most protective when consuming 22 g/day alcohol (RR 0.87) and became deleterious at just over 60 g/day alcohol (1.01 [0.71–1.44]). Among women, consumption of 24 g/day alcohol was most protective (0.60) and became deleterious at about 50 g/day alcohol (1.02) [0.83–1.26]).	20 cohort studies	Meta-analysis	Baliunas et al., 2009 [105]
Fasting plasma glucose is lower among drinkers compared to abstainers (97.6 ± 18.2 vs. 118.4 ± 29.6 mg/dL; *p* < 0.02). HDL cholesterol is significantly higher among drinkers compared to abstainers (46.9 ± 10.9 vs. 39.5 ± 9.0 mg/dL; *p* < 0.001).	101 moderate red wine drinkers and 104 abstainers	Case–control study	Rochitte et al., 2014 [106]
Compared with T2D patients who reported no alcohol consumption, those who reported moderate consumption had fewer cardiovascular events (adjusted hazard ratio [aHR] 0.83; 95% CI 0.72–0.95; *p* = 0.008), fewer microvascular complications (aHR 0.85; 95% CI 0.73–0.99; *p* = 0.03) and lower all-cause mortality (aHR 0.87; 96% CI 0.75–1.00; *p* = 0.05). The benefits were particularly evident in T2D participants who drank predominantly wine (cardiovascular events aHR 0.78, 95% CI 0.63–0.95, *p* = 0.01; all-cause mortality aHR 0.77, 95% CI 0.62–0.95, *p* = 0.02).	3314 patients with type 2 diabetes (T2D) who died or had CV problems during a 5 year follow up	Prospective cohort study	Blomster et al., 2014 [109]

### 5.3. Low-to-Moderate Wine Consumption and Neurodegenerative Diseases

This research was conducted with the keywords: “alcohol” AND “wine” AND “neurodegenerative diseases”. We analyzed a total of three studies: two population-based prospective study and one meta-analysis. The description of the studies is presented in Table 3.

Chronic alcohol abuse results in significant activation of neurodegenerative processes [111], but three out of the four selected studies reported in Table 3 show that the risk of developing neurodegenerative diseases is reduced in adult subjects with moderate consumption of alcohol beverages, in particular of wine [112,113,114]. Dementia and depression may be reduced by moderate wine drinking [112]. It has been suggested that low-to-moderate alcohol consumption could be beneficial to the health of middle-aged [115] and older subjects [116,117], leading to a J-shaped or inverse U-shaped association between alcohol and cognitive function [118]. The relative risks of developing ischemic stroke and Alzheimer’s disease or vascular dementia are also lowered by moderate alcohol consumption [119]. A fourth selected study [120] reported a J-shaped relationship between alcohol intake and cognitive decline in patients with mild cognitive impairment. This study showed that high consumption of alcoholic beverages, as well as complete abstention, increases the risk of dementia, while light–moderate alcohol drinking may be associated with a decreased risk of dementia [120]. In particular the study reported that, in adults, light-to-moderate and regular wine drinking has a protective effect against dementia [120]. These findings are confirmed by the review of Collins et al. [121]. A recent study confirms the previous data showing a protective effect of wine consumption against cognitive decline: the pooled RR for the effect of wine consumption on cognitive decline was 0.72 [113]. In 1998, the Copenhagen City Heart Study found a U-shaped relationship between intake of alcohol and risk of stroke. The intake of wine on a monthly, weekly or daily basis was associated with a lower risk of stroke compared with no wine intake (monthly: relative risk [RR], 0.83; weekly: RR, 0.59; daily: RR, 0.70). On the contrary, there was no association between intake of beer or spirits on risk of stroke [114] (Table 3).

**Table 3:** Studies on wine consumption and risk of neurodegenerative diseases.

Wine/Alcohol Consumption and Neurodegenerative Diseases	Number of Subjects	Study Design	References
Subjects drinking 3 to 4 standard glasses of wine per day (>250 and up to 500 mL), categorized as moderate drinkers, the crude odds ratio (OR) was 0.18 for incident dementia (*p* < 0.01) and 0.25 for Alzheimer’s disease (*p* < 0.03), compared to the non-drinkers. In the 922 mild drinkers (<1 to 2 glasses per day) there was a negative association only with AD, after adjustment (OR = 0.55; *p* < 0.05) vs non-drinkers.	922 mild drinkers, 318 moderate drinkers and 971 non drinkers	Population-based prospective study	Letenneur, 2004 [112]
The pooled RR for the effect of wine consumption on cognitive decline was 0.72 (95% CI 0.63–0.80; I2 = 82.4%; τ2: 0.0154). Using the Hartung–Knapp–Sidik–Jonkman method, the RR was 0.65 (95% CI 0.52–0.79; I2 = 94,531%; τ2: 0.057).	12 studies ranging from 360 to 10,308 subjects	Meta-analysis	Luceron-Lucas-Torres, 2022 [113]
Intake of wine on a monthly, weekly or daily basis was associated with a lower risk of stroke compared with no wine intake (monthly: relative risk [RR], 0.83; 95% CI, 0.69 to 0.98; weekly: RR, 0.59; 95% CI, 0.45 to 0.77; daily: RR, 0.70; 95% CI, 0.46 to 1.00). There was no protective association between intake of beer or spirits on risk of stroke.	13,329 eligible men and women, aged 45 to 84 years, participating in the Copenhagen City Heart Study	Prospective cohort study	Truelsen et al., 1998 [114]
Light–moderate alcohol drinkers had better MMSE (Mini Mental State Exanination) performance than abstainers (*p* < 0.05) and heavy drinkers (*p* < 0.01) 2 years after MCI diagnosis.	176 patients with mild cognitive impairment (MCI)	Prospective cohort study	Xu et al., 2009 [120]

### 5.4. Low-to-Moderate Wine Consumption and Cancer

This research was conducted with the keywords: “alcohol” AND “wine” AND “cancer”. We analyzed a total of five studies: one population-based multicenter prospective study, three prospective cohort studies and one case–control study. The description of the studies is presented in Table 4.

There is a strong scientific consensus that alcohol drinking can cause a dose-related increase of cancer risk [122]. Clear patterns have emerged between alcohol consumption and the development of cancer of head and neck, esophagus, liver, breast, colon and rectum [122]. As a consequence, many institutions and authorities provide guidelines and recommendations on alcohol consumption. The conclusions of The Third Expert Report “Diet, Nutrition, Physical Activity and Cancer: a Global Perspective” produced in 2018 by the World Cancer Research Fund International on behalf of the World Cancer Research [123] indicate that: “*for cancer prevention, it’s best not to drink alcohol. If you do consume alcoholic drinks, do not exceed national guidelines*”. The guidelines on alcohol and public health of the Centers for Disease Control and Prevention (CDC) of the United States of America [124] indicate that: “*The Dietary Guidelines for Americans recommend that adults of legal drinking age can choose not to drink, or to drink in moderation by limiting intake to 2 drinks or less in a day for men or 1 drink or less in a day for women*”. The recent paper by Clinton et al. [125] indicates that: “*1. consuming alcoholic drinks is a cause of several cancers. 2. for cancer prevention, it is best not to drink alcohol. 3. the evidence of increased cancer risk is for alcohol intake >30 g/day*”. Similar conclusions have been reported in the European code against cancer [126], which states in its 4th edition published in 2016: “*Not drinking alcohol is better for cancer prevention. If you drink alcohol of any type, limit your intake*” [127].

Resveratrol, found in grapes and wine (mostly red wine), has been investigated for many possible health effects, including cancer prevention [128]. Whether moderate intake of red wine may protect against cancer is still controversial [129]. The aforementioned health benefits of resveratrol against cancer produced in the laboratory setting cannot be translated into effects on the free-living population. Indeed, three cohort studies (conducted in non-Mediterranean countries) on lung, colorectal and prostate cancers, and one study on overall cancer mortality (conducted in a Mediterranean country) reported that moderate alcohol/wine consumption does not contribute appreciably to the etiology or protection of such malignancies (Table 4) [130,131,132,133].

The traditional Mediterranean diet that includes moderate wine consumption during adult life is associated with a reduced risk of cancer. This pattern of drinking does not appreciably influence the overall risk of cancer [134]. The only critical problem seems to be the breast cancer risk, but a recent study showed a strong association between adherence to the Mediterranean diet and reduction of breast cancer risk with an odds ratio of 0.82 [135] (Table 4). Interestingly, the exclusion of the ethanol component (mainly due to wine) from the Mediterranean diet score did not materially modify the results (OR = 0.81).

However, as previously said, heavy alcohol drinking is associated with digestive, upper respiratory tract, liver and breast cancers; therefore, restriction of alcohol consumption is a priority goal and the national and international authorities should suggest that alcohol consumption should be avoided to prevent cancer and when alcohol is consumed the amount should be limited to two drinks/day in men and one drink/day in women.

**Table 4:** Studies that considered wine and cancer risk.

Wine/Alcohol Consumption and Cancer	Number of Subjects	Study Design	References
In comparison with life-time abstainers, consumption of alcohol less than 10 g/day was associated with an average 11% [95% confidence interval (CI) = 7–14%] reduction in the risk of total mortality, while intake > 20 g/day was associated with a 13% (95% CI = 7–20%) increase in the risk of total mortality. With regard to cancer, drinking up to 10 g/day was not associated with either mortality risk reduction or increase, while alcohol intake > 20 g/day was associated with a 22% (95% CI = 10–35%) increased risk of mortality.	142,960 individuals (mean age 50 ± 13 years, 53.9% men)	Prospective observational multicenter population-based study	Di Castelnuovo et al., 2022 [133]
Compared to a Mediterranean diet score (MDS) of 0–3, the ORs for breast cancer were 0.86 (95% confidence interval, CI, 0.76–0.98) for a MDS of 4–5 and 0.82 (95% CI, 0.71–0.95) for a MDS of 6–9 (p for trend = 0.008). The exclusion of the ethanol component (mostly from wine) from the MDS did not materially modify the ORs (e.g., OR = 0.81, 95% CI, 0.70–0.95, for MDS ≥ 6).	3034 breast cancer cases and 3392 controls	Hospital-based case–control study	Turatti et al., 2018 [135]
Using men who did not consume red wine as the reference, no linear trend was observed between red wine consumption and prostate cancer in the full analytic cohort (*p*-trend = 0.57).	3348 cases of prostate cancer diagnosed among 45,433 eligible participants	Prospective cohort study	Sutcliffe et al., 2007 [132]
An inverse association between moderate red wine intake and risk of CRC was not found. The hazard ratio for consuming ≥ 1 drink /day (average = 2 drinks/day) was 1.16, 95% confidence intervals 0.56–2.40. There was no linear dose-response.	176 colorectal cancer patients diagnosed among 43,483 participants	Prospective cohort study	Chao et al., 2010 [131]
There was no clear association between lung cancer and consumption of beer, red wine, white wine or liquor at ≥1 drink/day. Alcohol intake at age 30 was not associated with lung cancer risk.	580 lung cancer cases diagnosed among 66,186 participants	Prospective cohort study	Chao et al., 2011 [130]

### 5.5. Low-to-Moderate Wine Consumption and Longevity

This research was conducted with the keywords: “alcohol” AND “wine” AND “longevity”. We analyzed a total of four studies: two population-based prospective studies, one longitudinal study and one meta-analysis. The description of the studies is presented in Table 5.

In all four selected studies the relationship between alcohol consumption and mortality is a J-shaped curve which shows that moderate consumption of alcohol reduces mortality compared to the absence of alcohol consumption [93,96,136,137] (Table 5). These benefits are observed at doses of approximately 3–30 g/day of alcohol in women and 12–60 g/day in men. A maximum protective effect is found at 20 g average of pure alcohol intake per day. The beneficial effects of moderate alcohol intake are greater than complete abstinence but are lost when consumption is excessive [102]. The Copenhagen Prospective Population Study demonstrated that wine intake may have a beneficial effect on all-cause mortality that is additive over alcohol alone [138] (Table 5).

Various studies have shown that resveratrol may prolong life span [139]. Indeed, resveratrol could increase longevity in many animal models, mainly by inducing Sirt1-dependent autophagy, reducing oxidative stress and neuroprotection [140], but additional research in humans is needed before drawing final conclusions on the correlation between resveratrol and longevity.

**Table 5:** Studies on wine consumption and longevity.

Wine/Alcohol Consumption and Longevity	Number of Subjects	Study Design	References
Compared with non-drinkers, light drinkers who avoided wine had a relative risk for death from all causes of 0.90 (95% CI, 0.82 to 0.99) and those who drank wine had a relative risk of 0.66 (CI, 0. 55 to 0.77).	13,064 men and 11,459 women, 20 to 98 years of age	Pooled cohort studies	Gronbaek et al., 1995 [95]
For each 2-point increment in a 0–9 score of adherence to the Mediterranean alcohol drinking pattern (MADP), a 25% relative risk reduction in mortality was found.	18,394 participants followed up to 12 years	Prospective cohort study	Gea et al., 2014 [102]
The pooled relative mortality risks were 0.90 (95% confidence interval: 0.81, 0.99) for 1–29 g/day of alcohol, 1.19 (95% confidence interval: 0.89, 1.58) for 30–59 g/day and 1.52 (95% confidence interval: 0.78, 2.98) for 60 or more g/day compared with abstention.	9 cohort studies 62,950 participants and 10,490 deaths	Meta-analysis	Jayasekara et al., 2014 [137]
Stable drinkers showed a U-shaped all-cause mortality, with relative risks of 1.29 (95% confidence interval [CI] = 1.13–1.48) for non-drinkers (<1 drink per week) and 1.32 (1.15–1.53) for heavy drinkers (>13 drinks per week) compared with light drinkers (1 to 6 drinks per week) For coronary heart disease mortality, stable nondrinkers had a relative risk of 1.32 (0.97–1.79) compared with stable light drinkers and those who had reduced their drinking from light to none increased their risk (1.40; 1.00–1.95), and those who had increased from nondrinking to light drinking reduced their relative risk ratio (0.71; 0.44–1.14).	6644 men and 8010 women, age 25 to 98 years, who had attended at least two health surveys with a 5-year interval between them	Longitudinal study	Gronbaek et al., 2004 [138]

## 6. The Mediterranean Way of Drinking: Wine in Moderation

Plant-based diets, defined in terms of low-to-moderate consumption of foods of animal origin, offer a wide range of health benefits, including aiding in the prevention and management of various diseases such as cardiovascular disease, diabetes, obesity and cancer [141]. The Mediterranean diet, a plant-based diet and one of the healthiest and most studied dietary patterns, includes two fluid foods in its nutritional guidelines: olive oil, as the main source of fat, and a low-to-moderate consumption of wine [142]. The mechanisms driving the beneficial effects of the Mediterranean diet include reduction of markers of inflammation and oxidative stress, improved lipid profile, insulin sensitivity and endothelial function, and antithrombotic properties. Most of these effects are attributable to bioactive ingredients, including fiber, polyphenols, and mono- and polyunsaturated fatty acids [142]. Moderate wine consumption is part of the Mediterranean diet, along with a “Mediterranean way of drinking”, i.e., regular and moderate consumption of wine in adulthood, mainly with food (up to two glasses a day for men and one glass for women) [2]. Wine is therefore considered a distinctive beverage of the Mediterranean diet that contributes to its health benefits, with some of the suggested biological pathways coinciding with those of the Mediterranean diet [143].

The proposed Mediterranean style of consumption [2] consists of a recommended limit of alcohol consumption that should not exceed 30 g of ethanol (i.e., about two glasses of wine per day, in combination with meals) for men and 15 g (one glass) for women. However, the definition of moderate alcohol consumption may vary from country to country. The United States, for example, considers moderate alcohol consumption by adults of legal drinking age to be a daily amount of 10 g ethanol (1 drink) for women and 20 g ethanol (2 drinks) for men. However, other guidelines consider daily consumption of 10 g to 42 g of alcohol (1 to 3 drinks) for women or 10 g to 56 g for men (1 to 4 drinks) to be a low-risk pattern [36].

## 7. The Impact of Alcohol Consumption on Human Health

Ethanol is a toxic and psychoactive substance with dependence producing properties due to a combination of genetic predisposition and brain damage from alcohol consumption. Approximately 4% of adults suffer from alcohol dependence, a complex behavioral syndrome involving an inability to control alcohol consumption despite known negative social, occupational and health consequences. Alcohol consumption is associated with health risks, even if these risks vary significantly in terms of magnitude and health consequences among drinkers, depending on the volume of alcohol consumed over time, type of alcoholic beverage, drinking pattern and diet.

Moreover, Stockwell et al. report that estimates of mortality risk from alcohol are significantly altered by study design and characteristics. Meta-analyses adjusting for these factors find that low-volume alcohol consumption has no net mortality benefit compared with lifetime abstention or occasional drinking [144]. In addition, the authors of the investigation of a large cohort from the European Prospective Investigation into Cancer concluded that various biases could influence the results of observational studies on the health benefit of low-to-moderate alcohol use [145].

Particular attention must be paid to young people. The Global Burden of Disease analysis estimates that, for young adults aged 15–39 years, alcohol consumption has no health benefits, only risks [146,147]. Alcohol is in fact the leading risk factor for premature mortality and disability among those aged 15 to 49 years, accounting for 10 percent of all deaths in this age group [148]. For adolescents and young adults, alcohol is the most widely used psychotropic substance and constitutes a serious health problem. The disease burden, social costs and harms associated with its use are extensive and common in young adults. Moreover, the development of subsequent alcohol-related harm has been linked to the early onset of alcohol consumption during adolescence [149]. Underage drinking is associated with a wide range of negative consequences for adolescents, including adverse effects on normal brain development and cognitive functioning, risky sexual behavior, physical and sexual assaults, injuries, blackouts, alcohol overdose and even death. When compared with use by adults, alcohol use by adolescents is much more likely to be episodic and in larger volumes (binge drinking), which makes alcohol use by those in this age group particularly dangerous [150].

Although excessive alcohol consumption is a serious public health problem worldwide due to its high prevalence and its health and social consequences, moderate intake, especially during meals, might be a low-risk model, but only for people over 40 years of age, as indicated by GBD 2020. For people in this age group, moderate alcohol intake may provide some health benefits, such as reduced risk of cardiovascular disease, stroke and diabetes, but also a possible increased risk of other diseases [146,147].

Ethanol is actually teratogenic, genotoxic and carcinogenic, hepatotoxic, neurotoxic to the brain, causes injury and increases the risk of cardiovascular disease and other noncommunicable diseases (i.e., HIV, TB, pneumonia and COVID-19 infection) [151].

Hence the need for educational programs on conscious consumption aimed especially at young people to avoid alcohol excess and abuse.

## 8. Conclusions

Wine is actually an alchemy of unique properties, with a rich and original composition in terms of polyphenols and antioxidants and a protective association between low-to-moderate wine consumption and cardiovascular diseases, type 2 diabetes and neurological disorders [152]. There is therefore strong scientific evidence from Mediterranean and non-Mediterranean countries that moderate wine consumption increases longevity, reduces the risk of cardiovascular disease and does not appreciably influence the overall risk of cancer [134] even though it has to be underlined that not drinking alcohol is better for cancer prevention [126].

The alcohol content varies between different types of wine, hovering around 14% for red wine and 11% for white wine, which is much lower than that of spirits (around 35%). Red wine has a high concentration of polyphenolic compounds; the content in white wine is lower, while it is practically negligible in distilled beverages (spirits and liqueurs) [152]. In addition to polyphenols, there may also be other phenolic and nonphenolic bioactive components in wine, usually less considered, that may contribute to the alleged health effects.

Bioactive components are not the only reason for the beneficial effects associated with wine consumption; social factors also matter. The Mediterranean diet is a dietary model that is also considered healthy because it suggests consuming wine during meals [2]. When consumed during meals, wine tends to be sipped more slowly than other alcoholic beverages and this may provide metabolic benefits. In addition, the concomitant presence of food in the stomach slows the absorption of ethanol, aiding metabolism and hepatic clearance, and lowering the peak blood alcohol concentration. The concomitant presence of food may also reduce the amount of alcohol available to the oral microbiota, which has the ability to metabolize ethanol to acetaldehyde, a compound associated with the tumor effects of ethanol in the upper gastrointestinal tract. In addition, the presence of alcohol may improve the bioavailability of polyphenols in the food bolus, making them more assimilable [153] and may reduce glucose bioaccessibility, which is consistent with the hypoglycemic effects observed in intervention and observational studies of moderate wine consumption.

This narrative review has limitations based on the literature selection, with a limited number of evaluated papers, and on the type of data analysis. Additional and future research with new meta-analyses of the existing data as well as new controlled studies and prospective studies have to be planned in order to analyze more precisely the existing data, to produce new evidence on this debated topic and to focus more clearly on the differential effects of wine versus other alcoholic beverages. Systematic reviews are needed to overcome the risks of bias of this paper and to define more clearly the negative and null literature data. In particular, this paper may show biases in paper selection and in outcome reporting.

This narrative review has been written to serve governments, organizations, industry, healthcare providers and individuals in a variety of capacities with the goal of improving health and reducing the global burden of various diseases, including cardiovascular diseases, type 2 diabetes and neurodegenerative diseases, and to favor longevity, but also to strongly remind of the negative effects of alcohol addiction.

In conclusion, wine differs from other alcoholic beverages and its moderate consumption not only does not increase the risk of chronic degenerative diseases but is also associated with health benefits. However, health care professionals should not recommend alcohol to nondrinkers because of the paucity of randomized outcome data and the potential for problem drinking even among individuals at apparently low risk, and every effort must be made to promote behavioral education to prevent abuse, especially among young people. Moreover, additional research is required to evaluate and clarify the doubts that still exist.

## Data Availability

No new data were created or analyzed in this study. Data sharing is not applicable to this article.
